# Antimicrobial activities of quaternary phosphonium-type small molecular antibacterial materials against methicillin-resistant *Staphylococcus aureus*

**DOI:** 10.1128/spectrum.00625-25

**Published:** 2025-10-29

**Authors:** Jing-Wen Deng, Si-Wen Deng, Jin-Huan Chen, Jin-Nan Wang, Hao-Hong Li, Yi Li, Jian-Zhi Liu

**Affiliations:** 1 Department of Otorhinolaryngology, Fujian Medical University Union Hospital 117890, Fuzhou, Fujian, People's Republic of China; 2 School of Medical Technology and Engineering, Fujian Medical University 74551, Fuzhou, Fujian, People's Republic of China; 3 College of Chemistry, Fuzhou University 12423, Fuzhou, Fujian, People's Republic of China; Guizhou Medical University, Guiyang, China

**Keywords:** quaternary phosphonium antibacterial material, methicillin-resistant *Staphylococcus aureus*, biocompatibility, antibacterial effectiveness, antibiofilm effect, antibacterial mechanism

## Abstract

Methicillin-resistant *Staphylococcus aureus* (MRSA) has become a significant public health concern due to its resistance to most antibiotics and its ability to form persistent biofilms, complicating treatment strategies. The need for new and effective antibacterial agents that can overcome these challenges is urgent. In this study, we address this issue by synthesizing and evaluating five quaternary phosphonium-type small molecular antibacterial agents ([MTPP]·I, [ETPP]·I, [ITPP]·I, [BTPP]·I, and [PTPP]·I) against MRSA. Our findings show that as the alkyl chain length of the phosphonium compounds increases, their antibacterial activity significantly improves, with [PTPP]·I exhibiting the highest efficacy. This compound demonstrated a minimum inhibitory concentration (MIC) of 16 µg/mL and was able to sustain bacterial growth inhibition for up to 36 h at the MIC. The study uniquely reveals a positive correlation between alkyl chain length and antibacterial potency, offering a novel insight into the mechanism of quaternary phosphonium salts. Mechanistic studies, including protein leakage assays, reactive oxygen species (ROS) measurement, and scanning electron microscopy (SEM), confirmed that [PTPP]·I targets MRSA by electrostatic interactions with the bacterial membrane, disrupting membrane integrity, inducing content leakage, and triggering ROS burst, ultimately leading to bacterial cell death. Additionally, [PTPP]·I demonstrated a 96.6% inhibition rate against MRSA biofilms and low cytotoxicity (cell viability >97%). This research not only presents a promising candidate for MRSA drug development but also contributes a comprehensive structure-activity-mechanism approach to combat bacterial resistance and biofilm-associated infections.

**IMPORTANCE:**

The rise of methicillin-resistant *Staphylococcus aureus* (MRSA) represents a significant threat to global health, largely due to its resistance to many antibiotics and its ability to form durable biofilms. This study presents a novel approach to combating MRSA through the development of a series of quaternary phosphonium-based antibacterial compounds. Among these, the compound with the longest alkyl chain ([PTPP]·I) stands out for its remarkable effectiveness, inhibiting MRSA growth at low concentrations, disrupting biofilms by 96.6%, and demonstrating minimal cytotoxicity to human cells. The research highlights the crucial relationship between alkyl chain length and antibacterial activity, providing a new strategy for designing safer and more potent antimicrobial agents. These compounds target bacterial membranes and overcome resistance mechanisms, offering a promising solution for treating MRSA infections and addressing the broader issue of antibiotic resistance, potentially saving lives and alleviating the global healthcare burden.

## INTRODUCTION

Antimicrobial resistance (AMR) is a growing global health crisis that threatens our ability to effectively treat bacterial infections. AMR has been ranked as the third leading cause of death in 2019, following ischemic heart disease and stroke, on the basis of the counterfactual of no infection (1, 2). The global spread of “superbugs” further exacerbates the concerning and urgent situation of drug-resistant pathogens. Methicillin-resistant *Staphylococcus aureus* (MRSA), a widely recognized “superbug,” has been associated with high morbidity and mortality rates resulting from antimicrobial-resistant infections worldwide (3, 4). Antibiotic resistance has far-reaching consequences, causing an estimated 700,000 deaths annually and projected to reach 10 million by 2050, with an economic loss of up to 100 trillion USD (5). The looming problem of AMR demands an urgent response with new novel antibiotics, yet, little effects have been obtained (6, 7). Therefore, effective molecules of both antibiotics and alternatives to antibiotics to fight drug-resistant pathogens need to be urgently explored.

Nanomaterials offer a promising solution to the issue of increased antibiotic resistance, which cannot be effectively tackled by conventional antimicrobial therapies (8). Nanomaterials refer to materials within the nano-scale range (1–100 nm) that exhibit unique physicochemical properties, including a high surface-to-volume ratio and distinct optical, magnetic, and electrical characteristics. These properties enable precise targeting mechanisms, thereby enhancing therapeutic efficacy while reducing adverse side effects (8, 9). Various nanomaterials, including silver-based (10, 11), gold-based (12, 13), and metal oxide nanomaterials such as zinc oxide (ZnO_2_) (14) and iron oxide (Fe_3_O_4_) (15), as well as nanocarriers referred to as “nanoantibiotics” (16) (e.g., mesoporous silica nanoparticles (17), have garnered significant interest due to their inherent antimicrobial properties and their potential to enhance the efficiency and safety of antibiotic use (13, 18). However, the toxicity of nano antibacterial materials cannot be ignored. Toxic effects of gold nanoparticles (AuNPs) may lead to a reduction in cell viability and the release of Ag in silver nanoparticles (AgNPs) may cause DNA damage (19). Furthermore, a recent study has shown that metal-based nanomaterials as antibacterial agents may accelerate AMR through a similar mechanism to the growth of resistance caused by misuse of antibiotics, such as horizontal transfer of antibiotic resistance genes (ARGs) (20).

Antimicrobial peptides (AMPs), as potential alternatives to antibiotics, have been attracting increasing interest for their application due to their excellent antibacterial properties, based on their biocidal activity, and their lesser likelihood of inducing drug resistance (21). Lee et al. reported that the powerful efficacy of AMP in reversing vancomycin-resistant *S. aureus* (VRSA) minimal inhibitory concentration (MIC) into susceptible ranges and demonstrated the ability to prevent biofilm production (22). What’s more, cationic antimicrobial agents quaternary heteronium salts (QHSs) such as quaternary phosphonium salts and quaternary ammonium salts have been designed and synthesized on the basis of antimicrobial peptides. Zhang et al. have constructed a multifunctional platform of chitosan quaternary ammonium salts (QCS)/polyvinyl alcohol (PVA)/polyethylene glycol (PEG) hydrogels (QPP), loaded with ZnO@CeO2 (ZC-QPP), for promoting wound healing (23). We have previously synthesized the quaternary phosphorus salt of (1,4-DBTPP)Br_2_, which exhibited effective therapeutic effects in a rat model of a superficial wound infected with MRSA (24). However, several challenges must also be addressed when considering the clinical application of antimicrobial agents, including enhancing their stability and reducing cytotoxicity (25).

In this study, we designed and successfully synthesized a series of quaternary phosphonium-type small molecular antibacterial compounds by elongating the length of alkyl groups, i. e., methyl-triphenyl-phosphonium iodide ([MTPP]·I), ethyl-triphenyl-phosphonium iodide ([ETPP]·I), *i*-propyl-triphenyl-phosphonium iodide ([ITPP]·I), *n*-butyl-triphenyl-phosphonium iodide ([BTPP]·I), and *n*-pentyl-triphenyl-phosphonium iodide ([PTPP]·I). We conducted systematic material characterizations on these materials and carried out assays to test *in vitro* antibacterial effectiveness, antibiofilm effects, antibacterial mechanisms, as well as cytotoxicity. The synthesized quaternary phosphonium salts ([MTPP]·I to [PTPP]·I) feature a central phosphorus atom covalently bonded to three hydrophobic phenyl groups and one alkyl chain (methyl to pentyl). The positively charged phosphorus atom facilitates electrostatic interactions with negatively charged bacterial membranes, while the hydrophobic alkyl chains enhance membrane penetration. The elongation of the alkyl chain increases lipophilicity, enabling deeper integration into bacterial membranes, thereby disrupting structural integrity. This dual mechanism—electrostatic attraction and hydrophobic disruption—underpins their potent antibacterial activity. This research aims to provide valuable insights for the development of similar antibacterial materials.

## MATERIALS AND METHODS

### Preparation of quaternary phosphoniums

**Fig 1:**
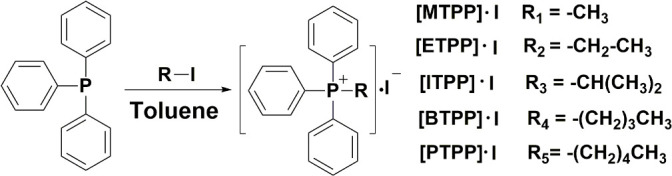
Process of the synthesis of five quaternary phosphonium molecules.

Five quaternary phosphonium molecules, i. e., [MTPP]·I, [ETPP]·I, [ITPP]·I, [BTPP]·I, and [PTPP]·I, were synthesized according to literature method, during which alkylated reactions of triphenyl-phosphine with iodomethane, iodoethane, *i*-propane iodide, *n*-butyl iodide, and iodopentane in the toluene have undergone (Fig. 1) (26). Setting [PTPP]·I as an example, triphenyl-phosphine (2.62 g, 10 mmol) was dissolved in 25 mL methylbenzene, and dropwise addition of iodopentane (2.4 g, 12 mmol) into the above solution was executed. The resultant mixture was kept reacting for 48 h at 120°C in a Schlenk tube. After cooling to room temperature, the solids were filtered with a Buchner funnel. The obtained white products were washed with iodopentane for three times and dried under vacuum (yield: 85%). Their ^1^H NMR spectra can be seen in Fig. S1.

### Bacterial culture

MRSA (ATCC 43300), *Escherichia coli* (*E. coli*, ATCC 25922), and *Pseudomonas aeruginosa* (*P. aeruginosa*, ATCC 27853) were purchased from ATCC (American Type Culture Collection, Virginia, USA) and used in related experiments. All bacteria cells were first pre-grown overnight on blood agar plates at 37°C in a bacteriological incubator. Next, a single colony was transferred into fresh Muller Hinton broth (MHB) medium and harvested by centrifugation during the exponential growth phase, before being resuspended in sterile 0.9% physiological saline. Finally, bacterial cells were adjusted to the 0.5 McFarland standard (1.5 × 10^8^ CFU/mL), and the solution was diluted in MHB to the predetermined concentrations.

### Cytotoxicity assay

The cytotoxicity of the nanocomposite was evaluated by CCK-8 assay using a commercial kit (CK04; Dojindo, Japan). L929 cells in DMEM (KeyGEN BiOTECH, Jiangsu, China) with 10% (vol/vol) fetal bovine serum were seeded into a 96-well microtiter plate at a density of 1.0 × 10^4^ cells/well, and at a final volume of 100 µL per well, the plate was placed in an incubator under 5% CO_2_, at 37°C for 24 h. Cells were then treated with [MTPP]·I, [ETPP]·I, [ITPP]·I, [BTPP]·I, and [PTPP]·I at the concentration of MIC. For another group, 10 µL of different concentrations of [PTPP]·I solution was added at the final concentrations of 8, 16, 32, 64, and 128 µg/mL to evaluate the dose-related cell viability of [PTPP]·I. After further incubation for 24 h, the solution was replaced by fresh medium excluding fetal bovine serum, and 10 µL of CCK-8 reagent was added per well, followed by incubation for another 2 h to detect the absorbance of the solution at 450 nm. Cell viability was calculated according to the following formula: cell viability (%) = (Ae − Ab)/(Ac − Ab) × 100% (Ab：OD of blank wells at 450 nm, Ac：OD of control wells at 450 nm, Ae：OD of experimental wells at 450 nm).

### Agar diffusion test

The inhibitory zone diameters of the five nanomaterials ([MTPP]·I, [ETPP]·I, [ITPP]·I, [BTPP]·I, [PTPP]·I) against three different strains (MRSA, *E. coli*, and *P. aeruginosa*) were performed with the agar diffusion test, which can visually present the antibacterial activity of nanocomposites, according to a previously reported method (27). Bacterial suspensions (1.5 × 10^8^ CFU/mL) were spread onto Mueller-Hinton (M-H) agar plates using sterile cotton swabs to form a uniform carpet. A 10 µL solution of each of the five nanomaterials at a concentration of 512 µg/mL, along with PBS, was carefully applied to the surface of filter paper discs (6.0 mm in diameter), which were then placed onto M-H agar plates and incubated overnight at 37°C. Inhibition zones appeared as transparent halos on the agar surface.

### The MIC test

MICs of all materials against MRSA, *E. coli*, and *P. aeruginosa* strains were determined using a modified broth microdilution method (28). Briefly, log-phase cultures (1.0 × 10⁶ CFU/mL) of each strain were inoculated into 96-well microtiter plates preloaded with serially diluted test compounds. After incubation at 37°C for 24 h, the MIC was defined as the lowest concentration that completely inhibited visible bacterial growth. The tested concentration ranges were from 4 µg/mL to 2,048 µg/mL for MRSA and *E. coli*, from 125 µg/mL to 64 mg/mL for *P. aeruginosa,* PBS and 2.5% DMSO served as controls.

### Bacterial growth curve study

Bacterial growth curve of [PTPP]·I against MRSA cells was determined by the method reported previously (29). MRSA cells (1.0 × 10^6^ CFU) were seeded in 100-well microtiter plates containing different concentrations at 1/8 × MIC, 1/4 × MIC, 1/2 × MIC, and 1 × MIC of [PTPP]·I and evaluated the optical density (OD 600) using an Automated Microbiology Analysis System (Bioscreen C, Turku, Finland) at 37°C with shaking at 200 rpm. The bacterial solution without [PTPP]·I was used as a positive control. OD values were continuously measured for 36 h at hourly intervals to construct bacterial growth curves.

### Time-kill kinetics assay

The bactericidal kinetics assay was carried out with a few minor modifications as previously stated to reveal the antimicrobial effectiveness of [PTPP]·I over time (30). Briefly, the log-phased bacterial culture was diluted with MHB to reach a concentration of roughly 1.0 × 10^5^ CFU/mL while being exposed to the different concentrations at 1 × MIC, 2 × MIC, 4 × MIC, and 8 × MIC of [PTPP]·I. The bacterial solution without [PTPP]·I was used as a control. Bacteria were sampled every 1 h from 0 h to 6 h, diluted in a gradient, and incubated in Luria-Bertani (LB) plates at 37°C for 24 h. Finally, plates with around 30 to 300 colonies were counted and CFU/mL for each time point was calculated to construct time-kill curves. After 3 h of treatment with different concentrations of [PTPP]·I, the bacterial solution was put into PBS for several consecutive 10-fold dilutions, and 100 µL of the mixture was uniformly spread on LB agar plates and then allowed to dry for a little while. The LB agar plates were placed in a constant-temperature incubator at 37°C for 24 h, and then the colonies on the LB agar plates were counted and photographed to observe the changes in the morphology and size of MRSA colonies.

### Scanning electron microscopy characterization of bacterial morphology and bacterial biofilm

Scanning electron microscopy was used to observe bacterial morphology and bacterial biofilm formation. After incubation with 0 and 32 µg/mL (2 × MIC) of [PTPP]·I for 24 h, bacterial cells were washed thrice with sterile 0.9% saline and then fixed in 2.5% glutaraldehyde for 2 h at 4°C. Samples were then washed with PBS and dehydrated in a graded ethanol series (30%, 50%, 70%, 80%, 90%, 95%, and 100%). After lyophilization and gold spraying, the MRSA cells were visualized by SEM (SU8100, Hitachi, Tokyo, Japan). Bacterial biofilms were subjected to SEM as previously reported with slight modifications (31). Cell crawling pieces were incubated in 1 mL of MRSA solution (1 × 10^6^ CFU/mL) in a 24-well plate. Experimental wells contained a solution of [PTPP]·I at a final concentration of 16 µg/ml (1 × MIC) and control wells did not. After 24 h of incubation at 37°C, the samples were processed as described above and the MRSA biofilms on the cell crawling pieces were observed by scanning electron microscopy.

### Crystal violet staining

Crystal violet staining was performed by the method reported previously to measure the anti-biofilm effect of [PTPP]·I (32). MRSA suspension (1 × 10^6^ CFU/mL) cultured in LB broth was added to 24-well plates containing [PTPP]·I at final concentrations ranging from 4 μg/mL to 64 μg/mL (i.e., 1/4 × MIC-4 × MIC) for 24 h at 37°C. A parallel study was also performed with LB broth as a blank control and the MRSA suspension was cultured in an empty well as a positive control. After incubation, the non-adhered bacteria were removed by gently washing with PBS at least three times, and the adherent biofilm was fixed with methanol for 15 min and stained with 0.1% (wt/vol) crystal violet for another 15 min. Then the excess crystal violet was removed by repeatedly washing with PBS until the blank control wells were colorless. Finally, crystal violet bound to the biofilm was dissolved in 500 µL 95% ethanol and added into 96-well plates to measure the absorbance at 595 nm using a Microplate Reader (Thermo Fisher Scientific, Waltham, MA, USA).

### Resazurin staining

The metabolic activity of bacteria within biofilms was tested using resazurin (Sigma Aldrich, St. Louis, MO, USA), as previous report with slightly modified (33). Bacterial suspensions were exposed to graded concentrations (range 4–32 μg/mL) of [PTPP]·I in MHB broth for 24 h at 37°C. After incubation, the wells were washed three times with PBS and 0.01% final concentration of resazurin (Sigma Aldrich, St. Louis, MO, USA) was added, followed by an incubation for 1 h. Finally, the absorbance was detected in a Multi-mode detection platform (Molecular Devices, SpectraMax i3x) in fluorescence mode with excitation at 570 nm and emission at 600 nm. Bacterial suspensions without [PTPP]·I were set as a positive control, and pure MHB broth was set as a blank control, which were taken as 100% and 0% metabolic activity, respectively. Metabolic activity was calculated according to the following formula: metabolic activity (%) = (ΔAbs of sample − ΔAbs of blank control)/(ΔAbs of positive control − ΔAbs of blank control); ΔAbs = absorbance at 570 nm − absorbance at 600 nm.

### Live/dead staining of Bacteria with SYTO 9/PI

MRSA cells were stained with a live/dead staining kit (SYTO 9 and propidium iodide) to perform a fluorescence observation referring to previous reports with minor modifications (34). The MRSA bacterial solution (1 × 10^8^ CFU/mL) and five different antimicrobial nanomaterials ([MTPP]·I, [ETPP]·I, [ITPP]·I, [BTPP]·I, [PTPP]·I) at a final concentration of 128 µg/mL were mixed in centrifuge tubes and incubated at 37°C. After 24 h, bacterial cells were collected by centrifugation (4°C, 5,000 rpm, 5 min), washed twice with PBS, and then stained with SYTO 9/PI staining kit. Cells were visualized using a fluorescence microscope (IX71, Olympus, Japan), and images were processed using imaging software (Image J).

### Protein leakage assay

The quantitative detection of protein leakage of bacteria was carried out by protein leakage assay using a Bradford Protein Assay Kit (P0006; Beyotime, Nantong, China). In brief, MRSA solution (1.0 × 10^8^ CFU/mL) was inoculated with [PTPP]·I at 32 µg/mL (2 × MIC) in 20 mL LB medium. The bacterial solution without [PTPP]·I was used as a control. The mixture was incubated in a shaker at 37°C for 24 h and then centrifuged at 10,000 rpm at 4°C for 10 min. The supernatant was immediately collected, and protein detection reagent was added to determine the optical density (OD 600) using a Microplate Reader (Thermo Fisher Scientific, Multiskan GO) and finally converted to protein concentration values based on the standard curve.

### Reactive oxygen species assay

The level of bacterial ROS induced by [PTPP]·I was detected by DCFH-DA assay (Reactive Oxygen Species Assay Kit, S0033S, Beyotime), as previously reported (18). Briefly, MRSA (1 × 10^8^ CFU/mL) was inoculated with 10 µM of DCFH-DA in a centrifuge tube at 37°C for 30 min for probe loading. After removal of the unloaded DCFH-DA by centrifugation, the MRSA cells were treated with different concentrations of [PTPP]·I (4 × MIC, 2 × MIC, and 1 × MIC) for 0.5 h. The bacterial solution without [PTPP]·I was included to use as a control. The samples were washed three times with sterile saline solution (0.9% NaCl). Finally, the level of bacterial total ROS was measured as the relative fluorescence intensity of the probes, which was detected in a Multi-mode detection platform (Molecular Devices, SpectraMax i3x) in fluorescence mode with excitation at 488 nm and emission at 525 nm.

### Statistical analysis

All quantitative experiments were performed with at least three independent biological replicates. The data presented in bar and line graphs correspond to the mean values derived from these independent biological replicates. Statistical significance was calculated using a one-way analysis of variance (ANOVA) (*P < 0.05).

## RESULTS AND DISCUSSION

### *In vitro* biocompatibility evaluation (cytotoxicity assay)

**Fig 2:**
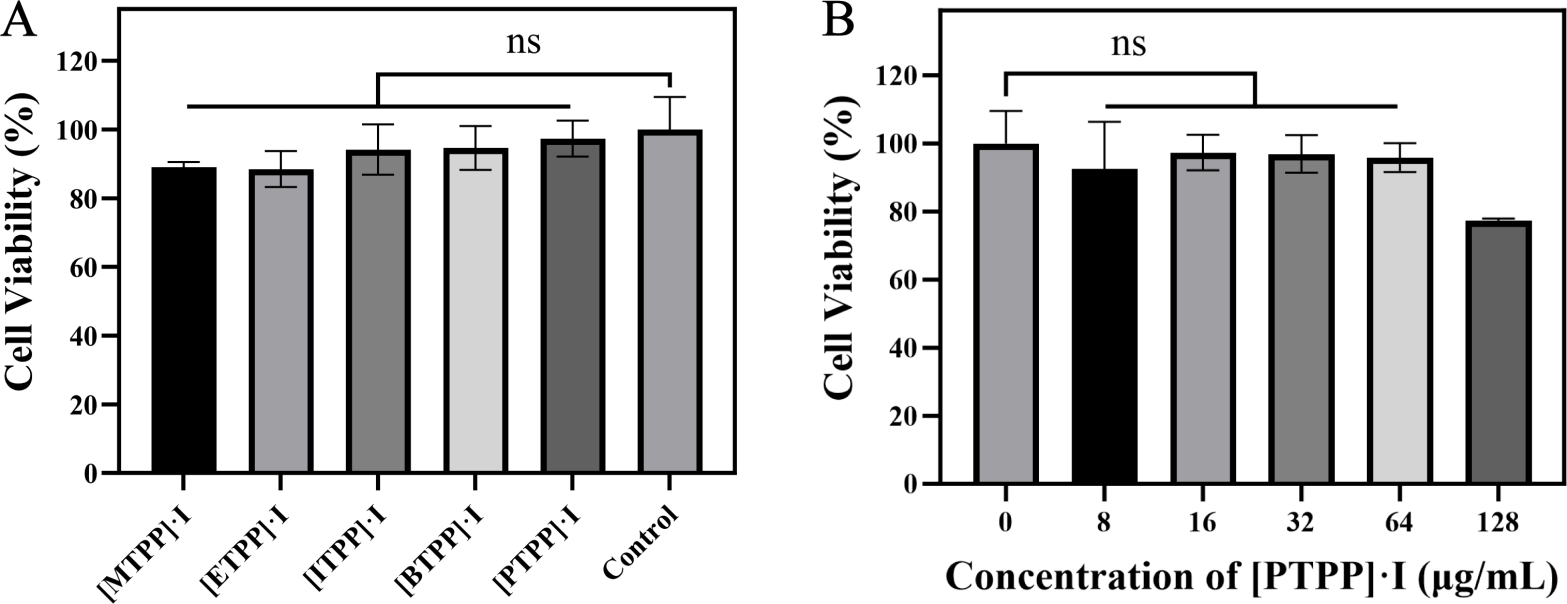
(**A**) Viability of L929 cells cultured with [MTPP]·I, [ETPP]·I, [ITPP]·I, [BTPP]·I, and [PTPP]·I at the concentration of MIC for 24 h. (**B**) Viability of L929 cells cultured with various concentrations (8, 16, 32, 64, 128 µg/mL) of [PTPP]·I for 24 h. Data are means ± SD, *n* = 3, ns, not significant (*P* > 0.05).

To evaluate the safety profile of nanocomposites, toxicity tests were performed on L929 cells. After incubation for 24 h, the cell viabilities of [MTPP]·I and [ETPP]·I remained ~90%, which is slightly lower than that for the three [ITPP]·I, [BTPP]·I, [PTPP]·I groups (~95%), indicating there were almost no cytotoxicity of nanocomposites on L929 cell line at the concentrations associated with MICs (Fig. 2A). In particular, the cell viability of [PTPP]·I was as high as 97% in the presence of MIC concentration, even at the high concentration of 128 µg/mL (8 times the MIC toward MRSA), cell viability remained ~80% after 24 h of stimulation (Fig. 2B). The results demonstrate that [PTPP]·I possesses better biocompatibility and a higher dose safety threshold than the other four nanomaterials.

### *In vitro* antibacterial activity of [PTPP]·I

**Fig 3:**
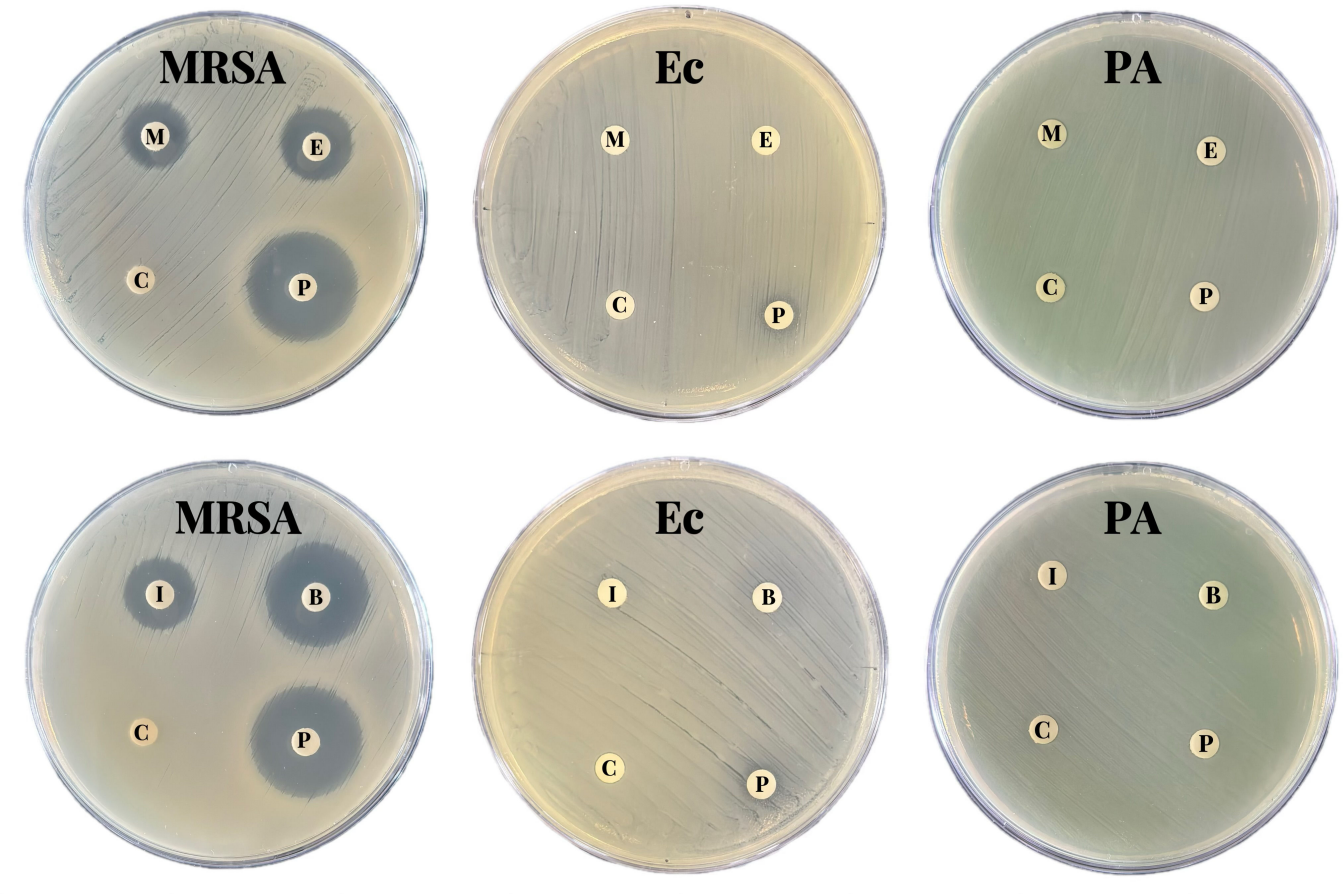
Digital images showing the inhibition halos caused by a series of quaternary phosphonium-type small molecular antibacterial materials against MRSA, *E. coli* and *P.aeruginosa*.(C: PBS, M: [MTPP]·I, E: [ETPP] ·I, I: [ITPP] ·I, B: [BTPP] ·I, P: [PTPP] ·I).

MIC is a key parameter for evaluating the susceptibility of microorganisms to antimicrobial, lower MIC indicates higher susceptibility and greater antimicrobial activity (35). At the same time, the determination of inhibition halos by agar diffusion test gives a good visual indication of the antimicrobial activity of the antimicrobial material. Therefore, we evaluated the zone of inhibition and MICs of the nanocomposites against three different bacterial strains (MRSA, *E. coli,* and *P. aeruginosa*). The MICs of the five nanocomposites for MRSA, *E. coli,* and *P. aeruginosa* are shown in Table 1. The MICs of [PTPP]·I were the lowest, with values of 16 µg/mL against MRSA, and the MICs of [MTPP]·I were the highest, with values of 128 µg/mL against MRSA. The MICs of the five nanocomposites were the same with values of 512 µg/mL against *E. coli*. The MICs of [MTPP]·I and [ETPP]·I were both 8 mg/mL against *P. aeruginosa*, but the MIC values of the three nanomaterials for *P. aeruginosa* were undetectable due to the solubility of [ITPP]·I, [BTPP]·I, and [PTPP]·I. The agar diffusion test (Fig. 3) further confirmed the stronger antibacterial activity of [PTPP]·I against MRSA; obvious inhibition halos appeared in the [PTPP]·I group, which were larger than those in the [MTPP]·I, [ETPP]·I, [ITPP]·I, and [BTPP]·I groups. The MIC for MRSA was lower than that for *E. coli* and *P. aeruginosa*, which might be caused by differences in cell wall composition between Gram-positive and Gram-negative bacteria. The cell wall of Gram-positive bacteria usually consists of only two layers, the peptidoglycan cell wall and the inner membrane. Gram-negative bacteria, on the other hand, have an outer membrane (OM) containing lipopolysaccharide/endotoxin in addition to the above two layers of structure, and most antibiotics need to pass through the OM in order to reach the target of drug action. The OM acts as a permeability barrier preventing antibiotics from penetrating the bacteria, which makes Gram-negative bacteria more resistant to antibiotics than Gram-positive bacteria; it has also been shown that any alteration of the OM, such as a change in hydrophobic properties or mutation of the pore proteins, can lead to the development of new resistance in Gram-negative bacteria (36–39). Therefore, it is crucial to develop novel antibiotics to overcome intrinsic and acquired resistance in Gram-negative bacteria. Furthermore, the antibacterial activity enhanced with the increase in chain length at carbon atom in the order [PTPP]·I > [BTPP]·I > [ITPP]·I ≥ [ETPP]·I > [MTPP]·I. The results are consistent with a previous report which revealed that structure-dependent activity of quaternary phosphonium salts in their antibacterial action against Gram-positive bacteria (40). As a result, [PTPP]·I showed the best antimicrobial efficiency against MRSA than the other four antimicrobial nanomaterials.

**TABLE 1:** Minimal inhibitory concentrations of a series of quaternary phosphonium-type small molecular antibacterial materials against MRSA, *E. coli,* and *P. aeruginosa^a^*

Strain	[MTPP]·I	[ETPP]·I	[ITPP]·I	[BTPP]·I	[PTPP]·I
MRSA (µg/mL)	≤128	≤64	≤64	≤32	≤16
*E. coli* (µg/mL)	≤512	≤512	≤512	≤512	≤512
*P. aeruginosa* (mg/mL)	≤8	≤8	/	/	/

^*a*^ /, MIC values for [ITPP]·I, [BTPP]·I, and [PTPP]·I against *P. aeruginosa* were not determined due to solubility limitations in the tested concentration range.

**Fig 4:**
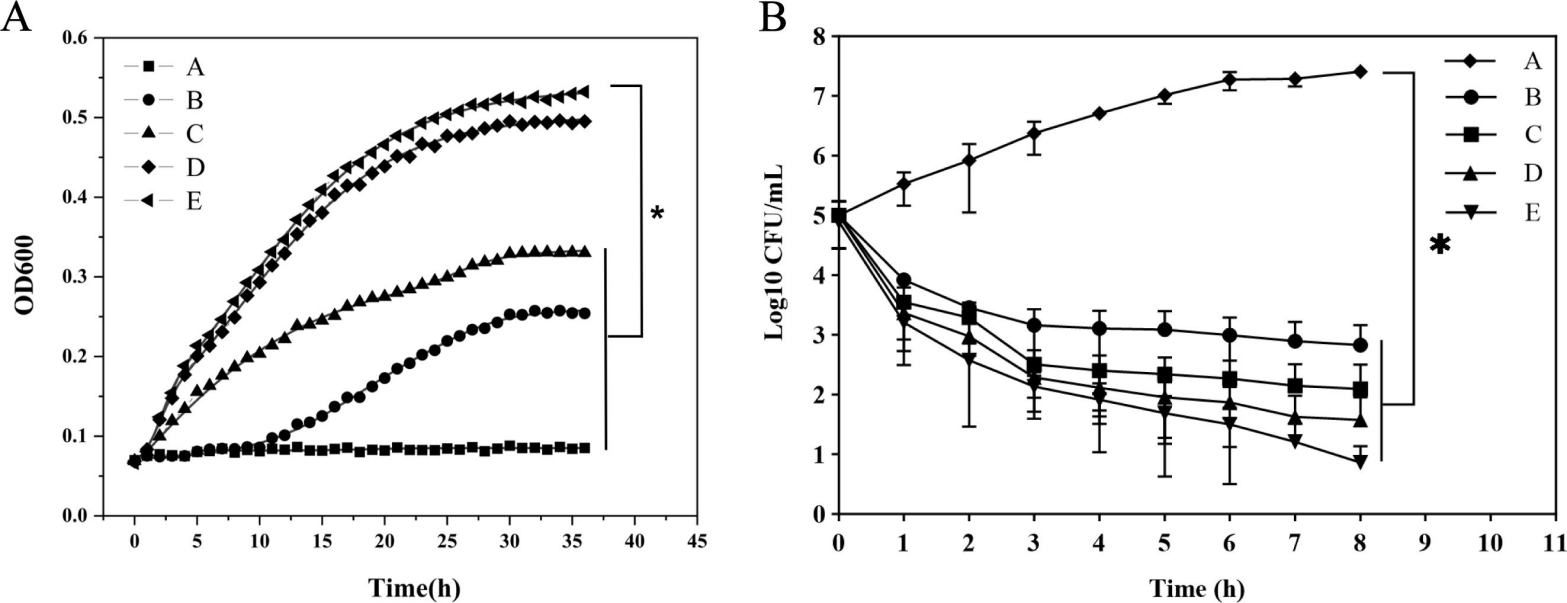
Antibacterial activity of [PTPP]·I against MRSA. (**A**) Growth curves of MRSA after incubation with various concentrations of [PTPP]·I (A–E∶1 × MIC, 1/2 × MIC, 1/4 × MIC, 1/8 × MIC, 0 × MIC). (**B**) Time-kill curves after incubation with various concentrations of [PTPP]·I (A–E: 0 × MIC, 1 × MIC, 2 × MIC, 4 × MIC, 8 × MIC). Data are means ± SD, *n* = 3, Student’s *t*-test; *P < 0.05 compared to the 0 × MIC group.

**Fig 5:**
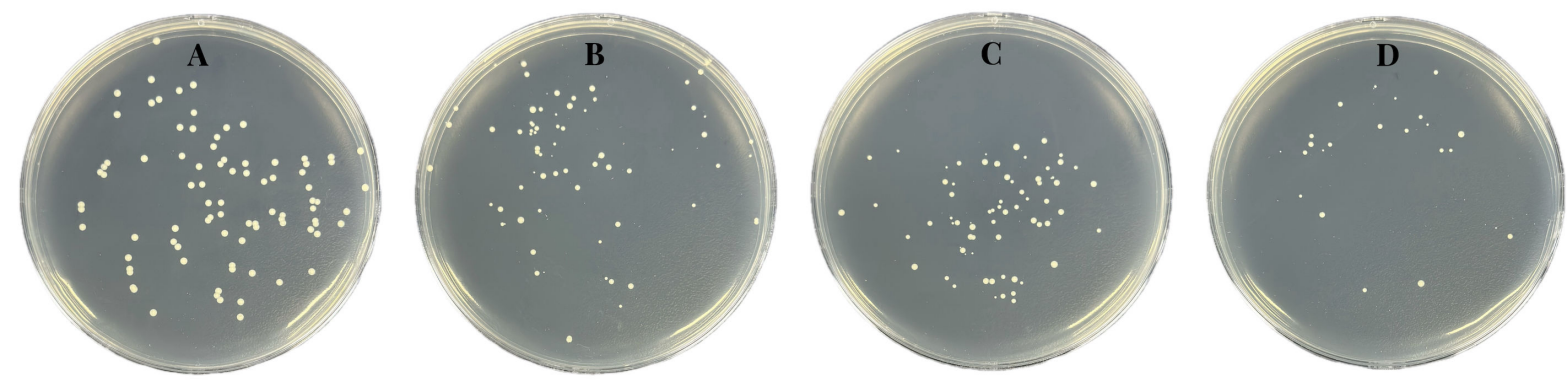
Changes in morphological size of MRSA colonies after treatment with different concentrations of [PTPP]·I for 3 h (A–D: 0 × MIC, 1 × MIC, 2 × MIC, 8 × MIC).

Next, we determined bacterial growth curve and time-kill curve to evaluate the *in vitro* antibacterial capability of the [PTPP]·I. The OD600 values of the MRSA solution were recorded every hour and measured continuously for 36 h under different concentrations of [PTPP]·I, and then, the fitted linear relationship was used to verify the results. As shown in Fig. 4A, bacterial growth can be significantly inhibited by [PTPP]·I at the concentrations of 2 µg/mL (1/8 × MIC) to 16 µg/mL (1 × MIC). [PTPP]·I at 1 × MIC displayed the strongest antibacterial effect, as OD600 values remain essentially unchanged for 36 h which means that there is almost no bacterial growth at MIC. As a consensus, bacterial growth in batch cultures occurs in four phases (lag, exponential/log, stationary, and death phase) (41). The effects of different antibacterial material on growth profiles varied but included increased lag phases and lethality, indicating both bacteriostatic and bactericidal activity (42). Against MRSA, [PTPP]·I at 1/2 × MIC caused an increased lag phase up to 10 h, which extended the lag phase but still allowed for bacterial growth, indicating bactericidal activity. Time-kill kinetics assay was further performed to evaluate the kinetic-killing effect of [PTPP]·I against the MRSA. Conventional colony counting remains the gold standard in microbiology to demonstrate bacterial viability (43). Consequently, time-kill curves obtained through colony counting method can effectively confirm the *in vitro* antibacterial efficacy of [PTPP]·I. The results constructed by plotting the log10 (CFU/mL) vs the incubation time are shown in the Fig. 4B. [PTPP]·I at 1 × MIC exhibited a time-dependent bactericidal activity against MRSA, resulting in an approximately 1-log_10_ CFU/mL reduction within 1 h (44). Besides, [PTPP]·I at 4 × MIC displayed a faster bactericidal effect relative to [PTPP]·I at 1 × MIC, as survival rate was less than 1% after treatment with [PTPP]·I at 4 × MIC for 2 h, while [PTPP]·I at 1 × MIC level took 6 h to achieve the same effect. These results are consistent with previous reports that [PTPP]·I induces bacterial cell death in a dose-dependent manner (24). Furthermore, colonies grown on LB plates (Fig. 5) showed that the viability of the treated bacteria, even if they survived, was greatly damaged, as evidenced by the uniform size of the untreated bacteria and the varying size of the [PTPP]·I-treated bacteria. Collectively, the bacteriostatic effect shown by growth curves and bactericidal effect shown by time-kill curves indicate that [PTPP]·I exhibits strong antibacterial activity against MRSA. To sum up, the highly effective antibacterial ability of [PTPP]·I, especially its excellent performance against the MRSA, indicates a great application potential of [PTPP]·I in the fight against the increasingly serious bacterial resistance problem.

### Antibiofilm formation effects by [PTPP]·I

**Fig 6:**
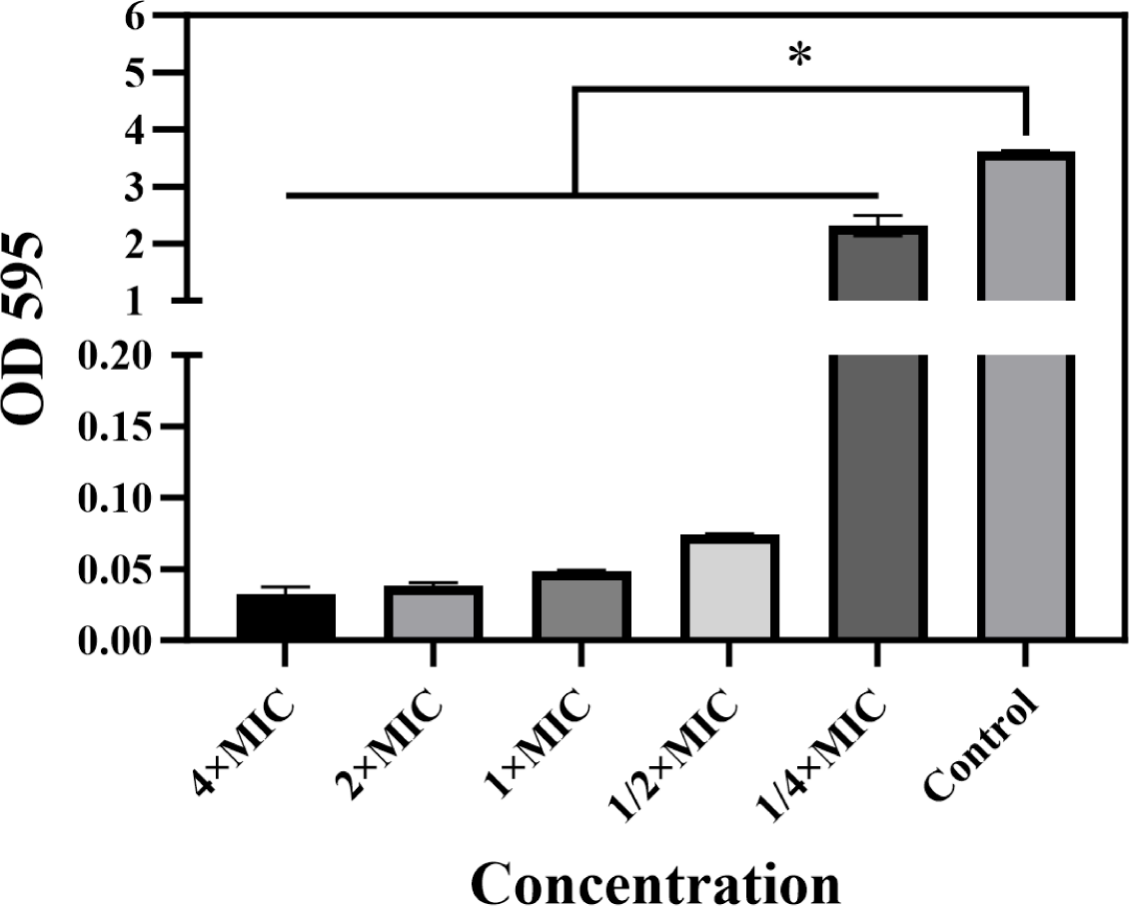
Crystal violet stain. (Quantification of [PTPP]·I on MRSA biofilm formation. Data are means ± SD, *n* = 3, *P < 0.05).

**Fig 7:**
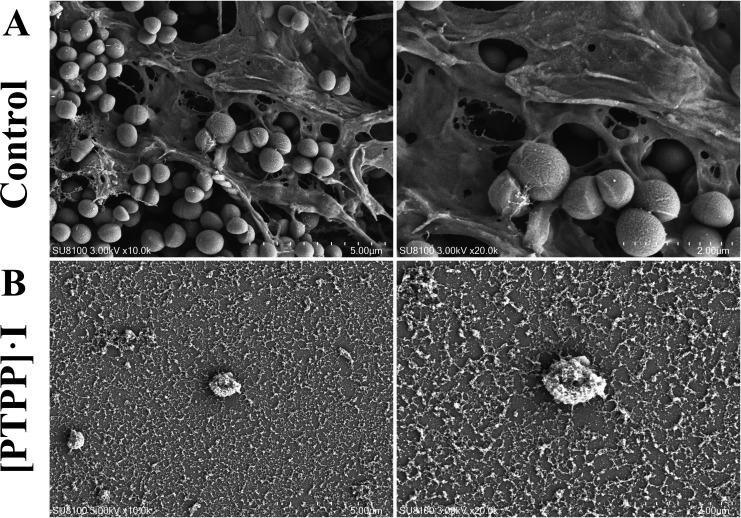
SEM images of MRSA biofilm: (**A**) control group, (**B**) [PTPP]·I treatment group.

**Fig 8:**
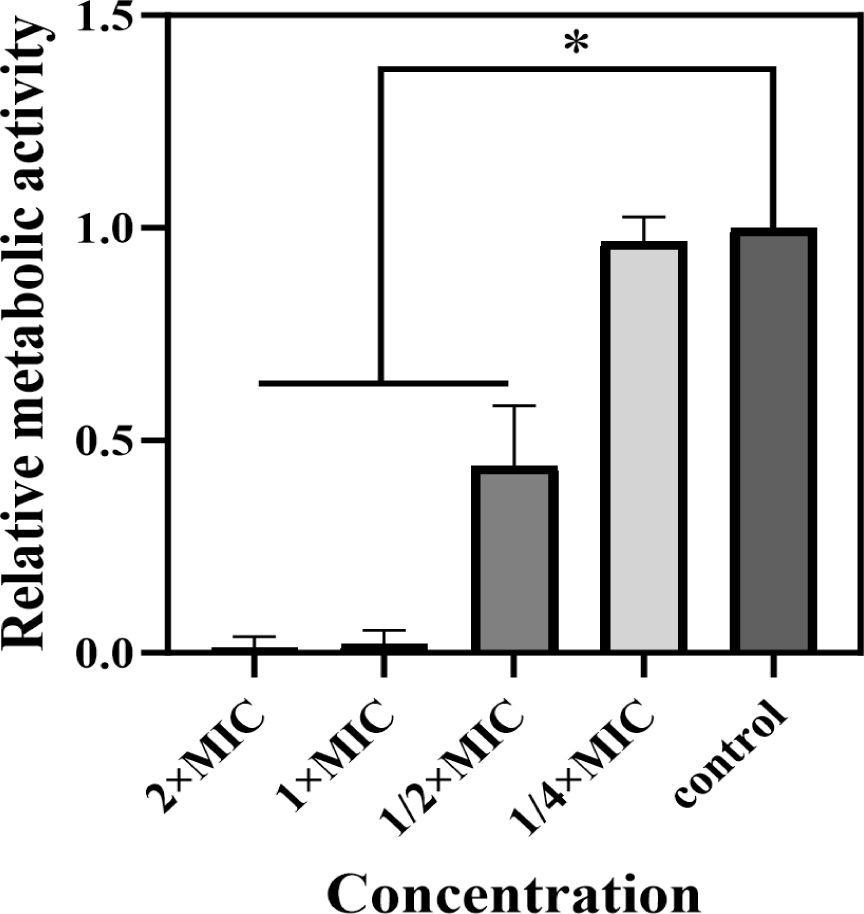
Resazurin stain. Quantification of [PTPP]·I on metabolic activity of bacteria within biofilms. Data are means ± SD, *n* = 3, *P < 0.05.

Biofilm refers to the complex communities of microbes. The biofilm matrix composed of extracellular polymeric substances (EPS) that, along with carbohydrate-binding proteins and other ingredients, act as a stabilizing scaffold for the three-dimensional biofilm structure (45, 46). Bacteria in biofilm are cocooned in a self-produced extracellular matrix, which makes them tolerant to harsh conditions and resistant to antibacterial treatments. Therefore, the anti-biofilm efficacy of antimicrobial materials is an important indicator for assessing their antimicrobial effectiveness. The crystal violet staining method was utilized to evaluate the impact of varying concentrations of [PTPP]·I on biofilm formation in MRSA. As shown in Fig. 6, the absorbance of stained biofilm in the control group was significantly higher than that of the [PTPP]·I groups, biofilm biomass decreased in a dose-dependent manner with [PTPP]·I. At the concentrations of 1/2 × MIC and 1 × MIC, [PTPP]·I significantly reduced the biofilm biomass of MRSA by 35.4% and 96.6%, respectively. Nevertheless, crystal violet assay may disrupt the biofilm due to its indirect nature and the need of repeated washings; SEM as a biofilm imaging technology has been proved to be very crucial to understand the complexity and dynamics of biofilms. To further visualize the biofilm-inhibition effect of [PTPP]·I, SEM analysis was conducted on MRSA biofilm. After 24 h of incubation, bacteria of the control group clustered around the secreted matrix, which indicated biofilm formation (Fig. 7A), and a large number of MRSA cells were wrapped in a biofilm on the cell crawling pieces. The SEM results revealed significantly dense and compact biofilms populated with an increased number of live cells following without [PTPP]·I treatment, whereas no biofilm but only the residue of bacterial disruption was observed on the cell crawling pieces following [PTPP]·I treatment (Fig. 7B). Collectively, these findings support those previously obtained (crystal violet staining) which provide strong evidence that biofilm formation was suppressed on the [PTPP]·I. In addition, the resazurin-based viability staining was used to quantify viable biofilm cells grown. Resazurin as an indicator of cellular metabolic ability is used to detect the activity of metabolism in cell cultures (47). This cell viability reagent is metabolized by bacterial dehydrogenases upon contact with living cells; as a result, the blue dye is reduced to a pink resorufin. As shown in Fig. 8, the metabolic activity of MRSA cells was significantly decreased by [PTPP]·I at the concentration from 1/2 × MIC to 2 × MIC. Exposure of MRSA biofilms to [PTPP]·I at 1/2 × MIC concentration reduced the metabolism of biofilm bacteria to more than 50% of the positive control, and increasing the concentration of [PTPP]·I to 1 × MIC further reduced the metabolic activity of intra-film bacteria to 1% of that of the positive control. The above three studies have demonstrated that [PTPP]·I can effectively inhibit biofilm formation by MRSA and significantly affect the metabolic activity of intramembrane bacteria. Bacterial biofilm development involves three phases: attachment, maturation, and dispersal (48). More work lies ahead to determine which phase of biofilm formation was influenced by [PTPP]·I.

### Antibacterial mechanisms of [PTPP]·I

**Fig 9:**
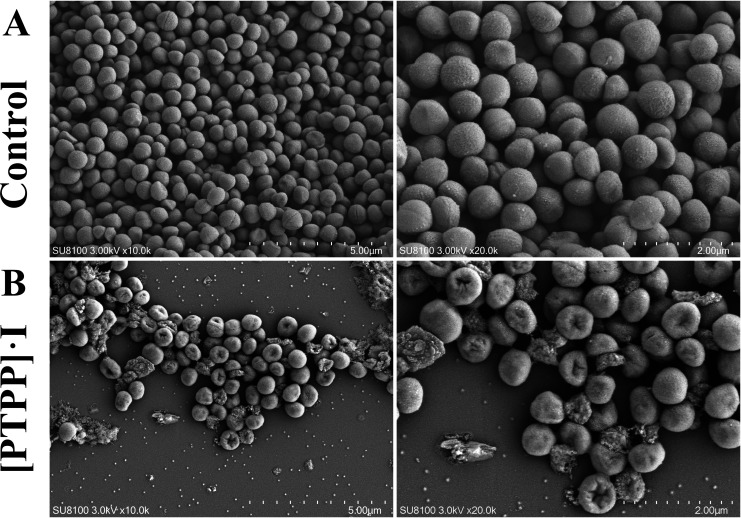
SEM images of MRSA suspended bacteria: (**A**) control group, (**B**) [PTPP]·I treatment group.

To explore the antibacterial mechanism of [PTPP]·I, SEM was used to observe bacterial morphology. Control MRSA cells are of normal shape as shown in Fig. 9, with smooth membranes/walls and cellular integrity. In contrast, after exposure to the [PTPP]·I, the membranes/walls of MRSA cells became rough and concave, holes appeared, and a large proportion of the bacteria even fragmented directly. This is consistent with a report showing that loss of membrane integrity caused by the antimicrobial material is likely responsible for the apoptosis of the bacterial cells (49).

**Fig 10:**
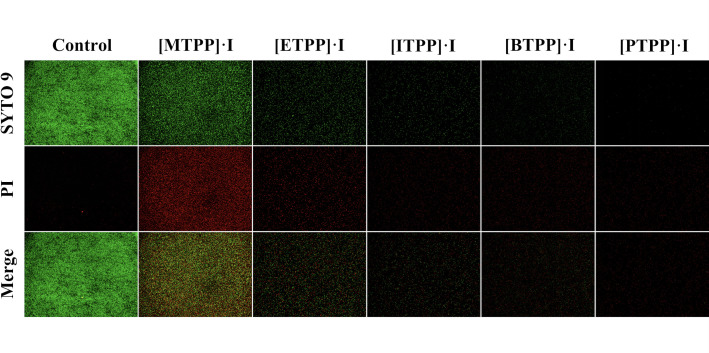
Fluorescence staining images of MRSA after treatment with 128 µg/mL of [MTPP]·I, [ETPP]·I, [ITPP]·I, [BTPP]·I, and [PTPP]·I. (SYTO 9: green fluorescence, staining both live and dead bacteria, PI: red fluorescence, staining dead bacteria).

Although SEM showed disruption of bacterial morphology by the [PTPP]·I, only a small number of MRSA cells could be seen in the field of view. To investigate the overall antibacterial effects, nucleic acids were labeled to determine the live/dead ratio using a green fluorescent membrane-permeable dye (stain both live and dead cells) and a red fluorescent dye propidium iodide (only stain dead cells) (50). As displayed by the green fluorescence in Fig. 10, bacterial cells were alive in the non-treated control group, and in the treatment groups, there were few live bacteria, indicating damage to the cell membranes/walls after exposure to antibacterial material. In the different treatment groups (in the order of [MTPP]·I, [ETPP]·I, [ITPP]·I, [BTPP]·I, [PTPP]·I), the proportion of green fluorescence showed a decreasing trend, indicating a gradual reduction in the number of live cells. This result indicates that the antimicrobial activity of the nanomaterials is enhanced with the increase in alkyl chain length, which was in accordance with the agar diffusion test and the MIC data. Moreover, after treatment with the equal dose (128 µg/mL) of nanocomposite, less bacteria were observed for [PTPP]·I than for the other four antimicrobial materials, suggesting that [PTPP]·I is better in terms of antimicrobial properties.

The existence of a correlation between efficiency of antimicrobial activities of quaternary phosphonium-type small molecular antibacterial materials and the length of the alkyl chain observed in this study is consistent with data previously published for some QHSs (51). The increase in the length of the alkyl chains from [MTPP]·I to [PTPP]·I led to a decrease in the MIC values, suggesting that the lengthening of the alkyl substituents increased the antimicrobial activity of quaternary phosphonium-type small molecular antibacterial materials, which can be explained by previous reports of structure-activity relationship studies on the effect of different alkyl chain lengths on antimicrobial activity, where the longer hydrophobic alkyl side chain of [PTPP]·I promotes strong interactions with bacterial membranes, which, in combination with the high permeability of the MRSA peptidoglycan cell wall to quaternary phosphonium-type small molecule antimicrobial materials, leads to the higher sensitivity of MRSA to [PTPP]·I (52).

**Fig 11:**
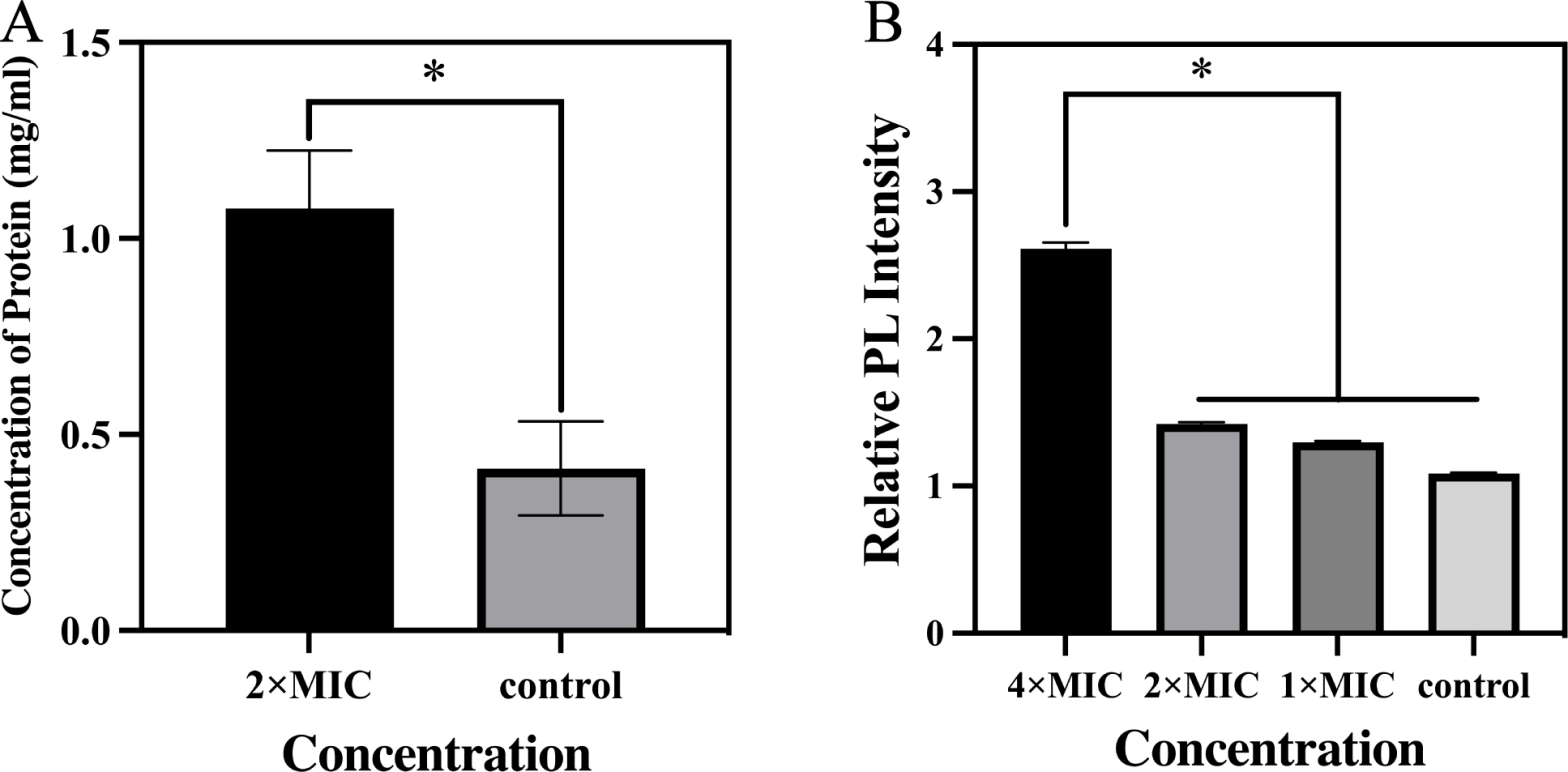
(**A**) Protein leakage from MRSA suspensions of [PTPP]·I treated group and control group. (**B**) Relative PL intensity of intracellular ROS production level in MRSA treated with various concentrations of [PTPP]·I for 0.5 h. Data are means ± SD, *n* = 3, *t*-test, *P < 0.05.

Next, protein leakage, as one of the representative indicators of cytoplasm leakage, is widely detected in verifying damage to bacterial structures. Therefore, we performed a quantitative protein leakage assay to confirm the destruction of the integrity of the membranes/walls of MRSA by [PTPP]·I. As shown in Fig. 11A, protein leakage in the [PTPP]·I group was about 2.6 times higher than that of the control group, which further confirms the destruction of bacterial structure.

In addition, the production of ROS as a common mechanism behind the activity of bactericidal antibiotics was confirmed (53). Except for destroying the integrity of the bacterial structure, the antibacterial efficacy of [PTPP]·I was also investigated on the basis of changes in ROS level. For MRSA cells, after treatment with 1 × MIC, 2 × MIC, and 4 × MIC concentration of [PTPP]·I, ROS levels were elevated by 20%, 30%, and 141% vs the control group, respectively (Fig. 11B), suggesting that [PTPP]·I promoted the production of intracellular ROS with dose dependence. ROS accumulation requires a higher stress threshold than early responses like membrane perturbation. As proposed by Zhao and Drlica, sub-lethal stress primarily activates protective systems, while ROS-mediated lethality occurs only when damage exceeds cellular repair capacity (54). This threshold effect aligns with Kohanski’s model where NADH depletion—triggered by sustained target inhibition—is essential for ROS generation (55). This is consistent with reports showing that higher concentrations of ROS lead to membrane degradation, metabolic hydroxylation, and oxidative DNA damage, resulting in higher microbial death (56, 57).

Our SEM showed microscopically that [PTPP]·I caused noticeable destruction to the membranes/walls of MRSA, and the large-scale damage in integrity of bacterial membranes/walls after treatment with [PTPP]·I was confirmed by the fluorescence microscopy. Furthermore, protein leakage further reconfirmed the disruption of cellular structure by [PTPP]·I. The bacterial cell membrane is a complex multilayered structure. In Gram-positive bacteria (e.g., Staphylococcus aureus), the outermost bacterial cell wall consists mainly of peptidoglycan, teichoic acids (TAs, including wall teichoic acids and lipoteichoic acids) and a variety of proteins. Phosphomimetic acid is rich in negatively charged phosphate groups, conferring significant negative electrical properties to the cell surface. The cell membrane (phospholipid bilayer) on the inside of the cell wall, on the other hand, contains mainly cardiolipin, phosphatidylglycerol, and lysyl-type phospholipids characteristic of *S. aureus* (58–60). The hydrophilic/hydrophobic nature of this composite structure, as well as the localized charge distribution properties, together determine its mode of interaction with external molecules, especially charged compounds. Since quaternary phosphonium-type small molecular antibacterial agents contain positively charged cations, they can bind to the negatively charged TAs in the bacterial cell wall through electrostatic interactions to neutralize the negative charge on the bacterial surface. At the same time, the hydrophobicity of the alkyl chains of quaternary phosphonium-type small molecule antimicrobial materials and their sizes match the hydrophobic core of the lipid bilayer of the cell membrane so that part of the hydrophobic alkyl chains can be embedded in the hydrophobic region of the lipid bilayer, and this embedding changes the physical state of the membrane, leading to an increase in the permeability of the membrane, which, in turn, may trigger the rupture of the membrane or the formation of pore channels, and ultimately destroy the integrity of the membrane, resulting in the leakage of the cytoplasmic contents (61). It should be noted that the antimicrobial efficacy of these compounds is related to the alkyl chain length, and compared with the other four quaternary phosphonium-type small molecule antimicrobial materials, the longer alkyl chain (e.g., pentyl group in [PPTP]·I), the stronger the effect on enhancing the hydrophobicity, which allows it to penetrate more deeply into the lipid bilayer of the bacterial membrane, thus exhibiting stronger antimicrobial activity (62). In terms of proposal, it is speculated that the antimicrobial process of [PTPP]·I may proceed as follows: first, the excellent electrostatic attraction promotes the positively charged [PTPP]·I to rapidly capture the negatively charged bacteria and then damage the bacterial cell, which causes the loss of cellular integrity with cytoplasm leakage and high levels of ROS, ultimately leading to bacterial apoptosis, which is consistent with previous reports (18, 63–65).

Ongoing research is focused on refining these compounds, particularly by molecular modifications to enhance the aqueous solubility, biocompatibility, and antimicrobial activity of [PTPP]·I. Based on literature analysis, introducing sulfonic acid groups is expected to enhance the antibacterial and anti-biofilm activity of [PTPP]·I. The incorporation of sulfonic acid groups can improve the polymer’s water solubility and electronegativity. It may also disrupt bacterial cell membrane integrity through electrostatic interactions and interfere with the secretion of extracellular polymeric substances (EPS) within biofilms, thereby effectively inhibiting bacterial adhesion and biofilm formation (66). Meanwhile, by compositing with biomass materials like chitosan, the quaternary phosphonium salt-sulfonic acid polymer can leverage chitosan’s inherent biocompatibility, biodegradability, and low toxicity to significantly enhance the material’s biosafety (67, 68). This composite strategy preserves the strong antibacterial efficacy of quaternary phosphonium salts while reducing potential cytotoxicity through the natural polysaccharide carrier. Moreover, comprehensive evidence indicates that strategic modulation of structural parameters—including alkyl chain length, cationic charge distribution, and lipophilicity—is expected to enhance antibacterial efficacy while maintaining low toxicity toward mammalian cells (52, 69, 70). In addition, our future work will also focus on *in vivo* validation using a murine MRSA infection model and synergy testing with conventional antibiotics (such as vancomycin). These studies will further determine the therapeutic potential of [PTPP]·I for treating drug-resistant infections, either as a standalone or adjunctive therapy.

### Conclusions

This study presents a series of quaternary phosphonium-type small molecular antibacterial materials, including [MTPP]·I, [ETPP] ·I, [ITPP]·I, [BTPP] ·I, and [PTPP]·I, as promising alternatives for combating MRSA. These compounds effectively inhibit bacterial growth and disrupt biofilm formation with minimal cytotoxicity. Notably, [PTPP]·I demonstrated significant efficacy against MRSA by suppressing planktonic bacterial activity and disrupting bacterial biofilms, suggesting its strong potential for clinical application.

## SUPPLEMENTAL MATERIAL

Figure S1 spectrum.00625-25-s0001.docx 1H NMR spectra of quaternary phosphonium molecules.

ASM does not own the copyrights to Supplemental Material that may be linked to, or accessed through, an article. The authors have granted ASM a non-exclusive, world-wide license to publish the Supplemental Material files. Please contact the corresponding author directly for reuse.
