# Supplementary material for: Antimicrobial activities of quaternary phosphonium-type small molecular antibacterial materials against methicillin-resistant Staphylococcus aureus
Source: Microbiol Spectr. 2025 Oct 29;13(12):e00625-25. doi: 10.1128/spectrum.00625-25 (PMC12671080; doi:10.1128/spectrum.00625-25)
Supplement: Figure S1 — 1H NMR spectra of quaternary phosphonium molecules. [file spectrum.00625-25-s0001.docx]

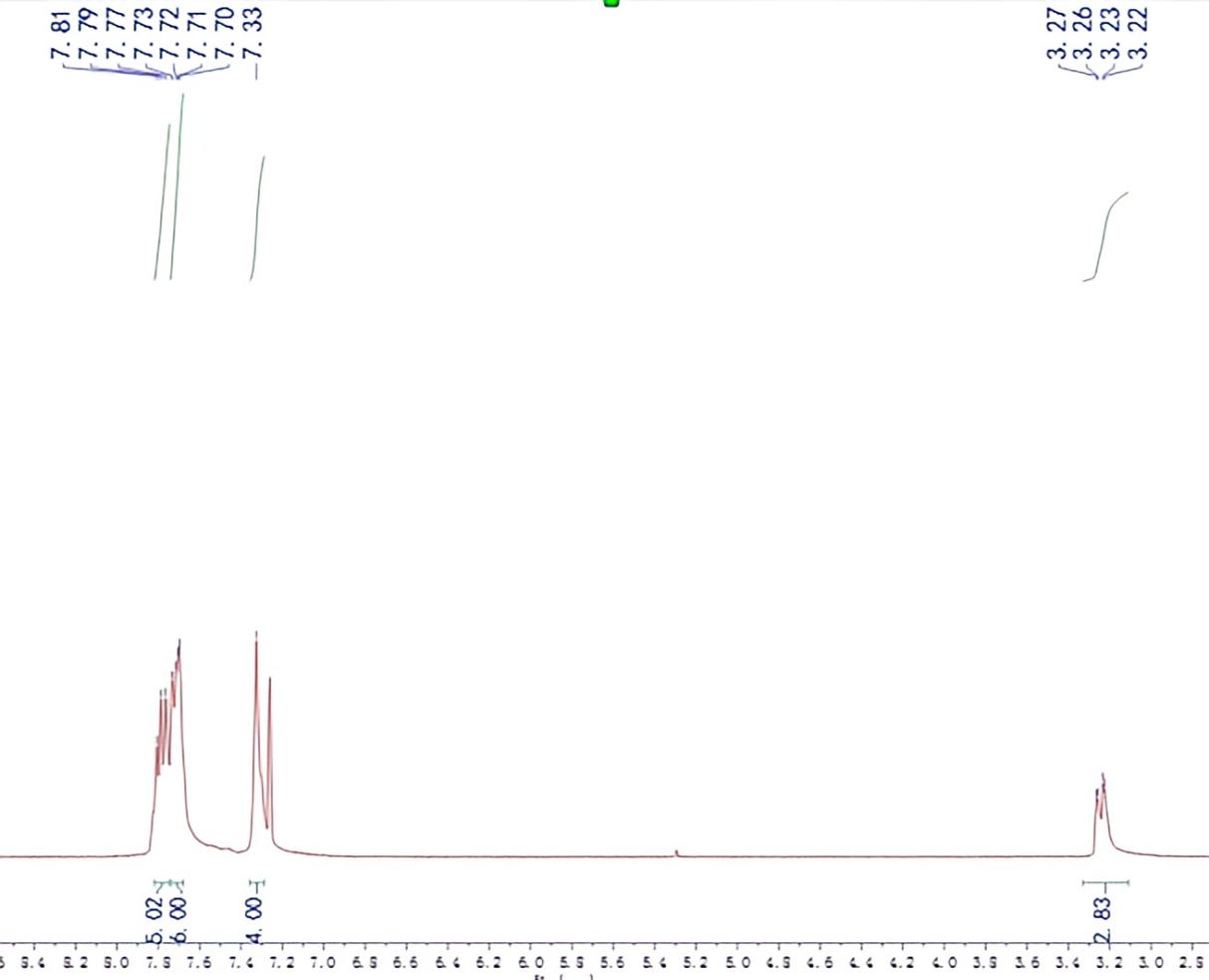


**(A)**

**
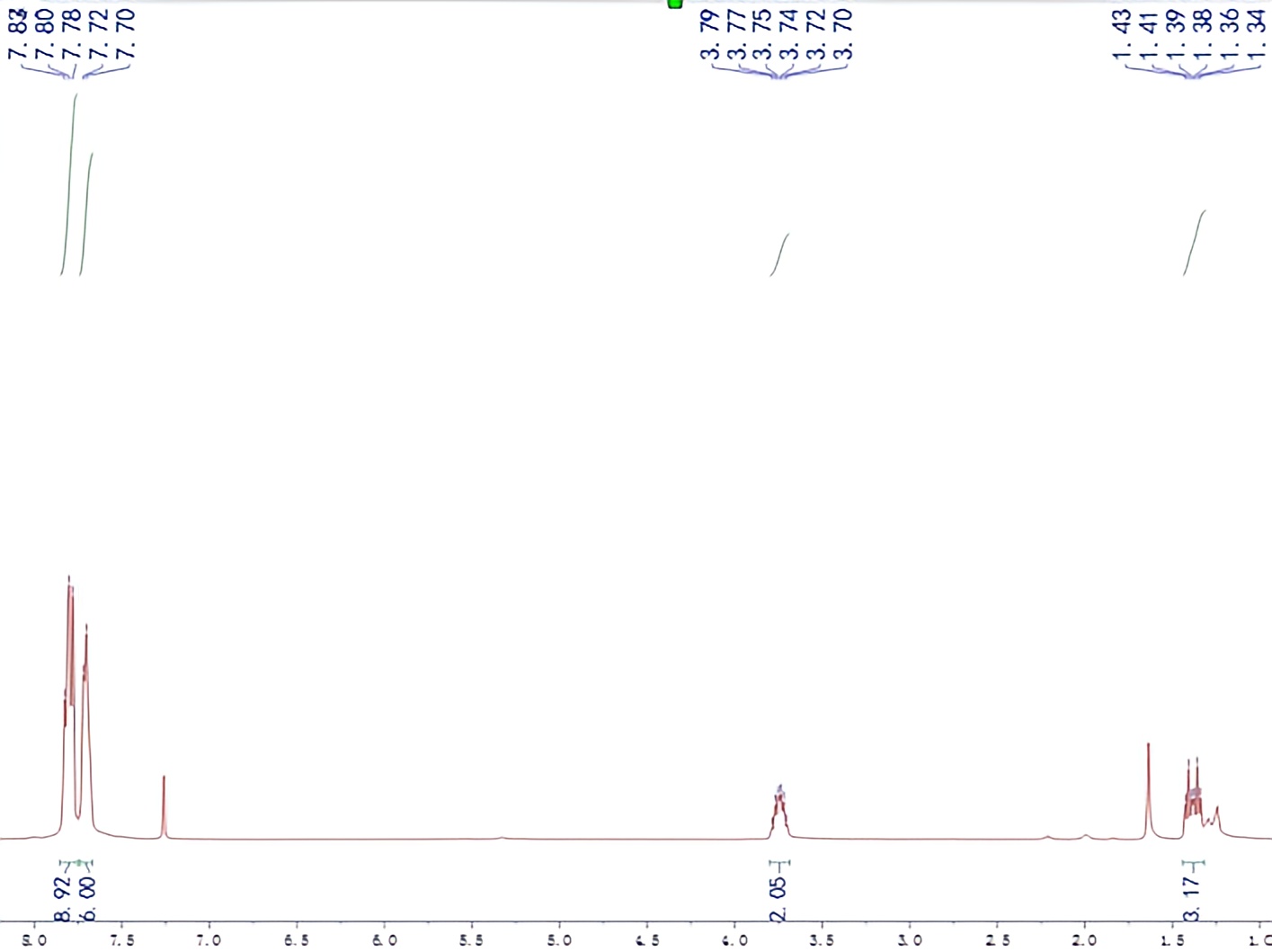
**

**(B)**

**
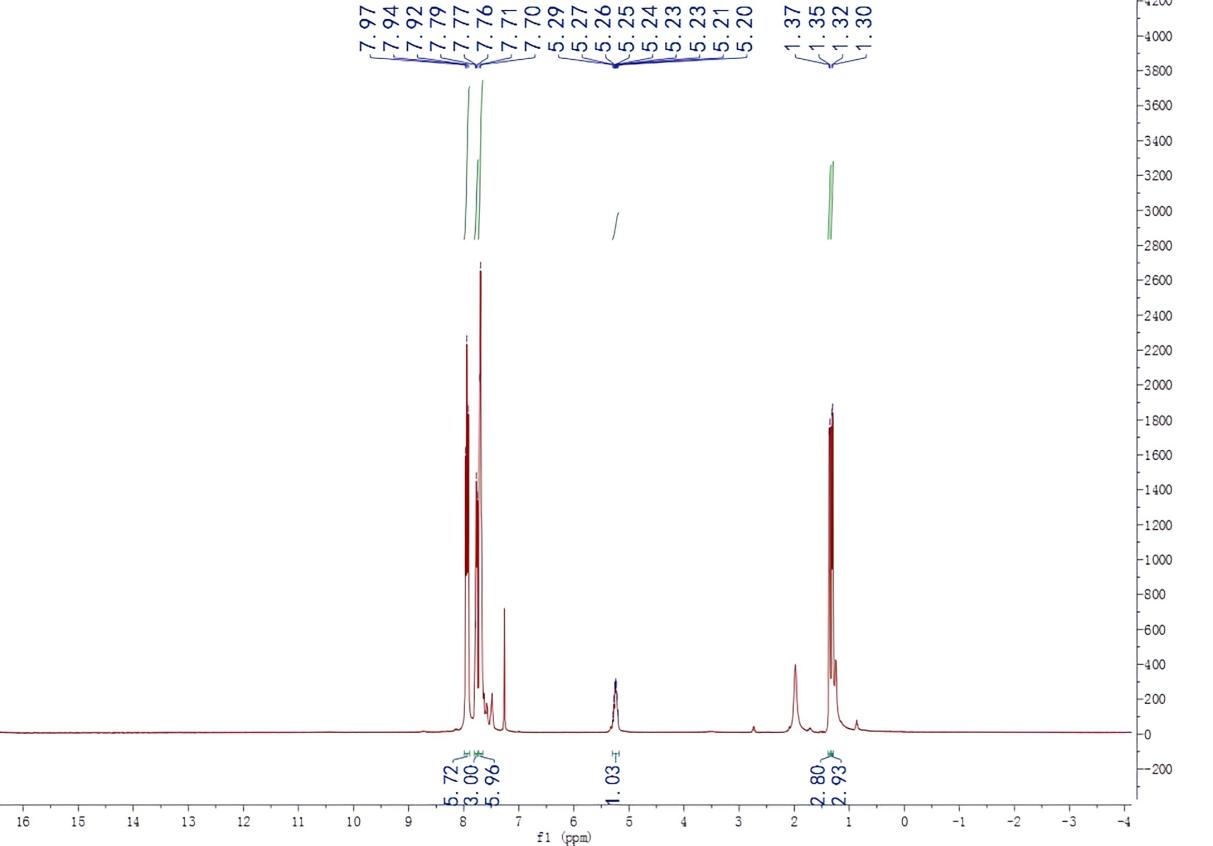
**

**(C)**

**
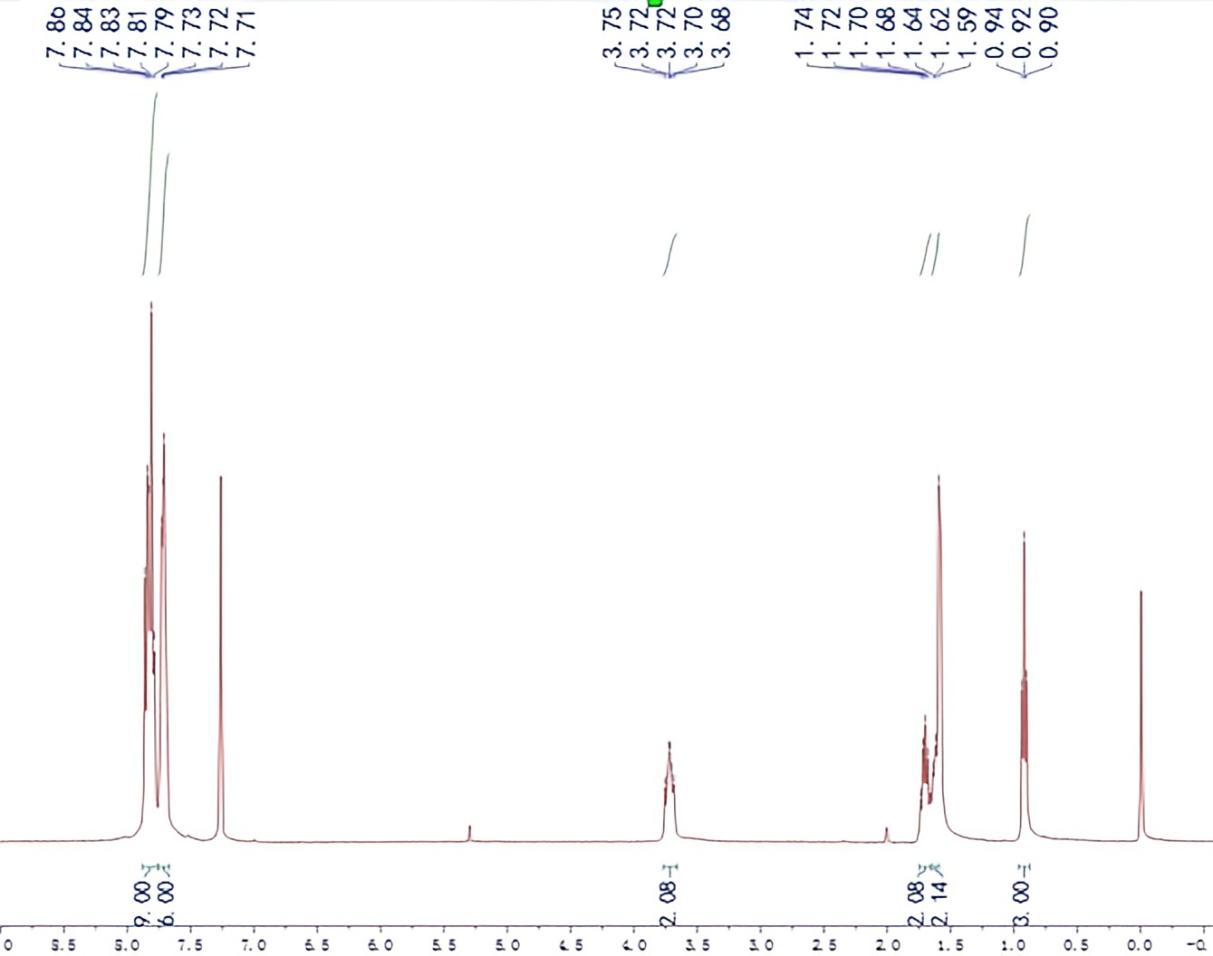
**

**(D)**

**
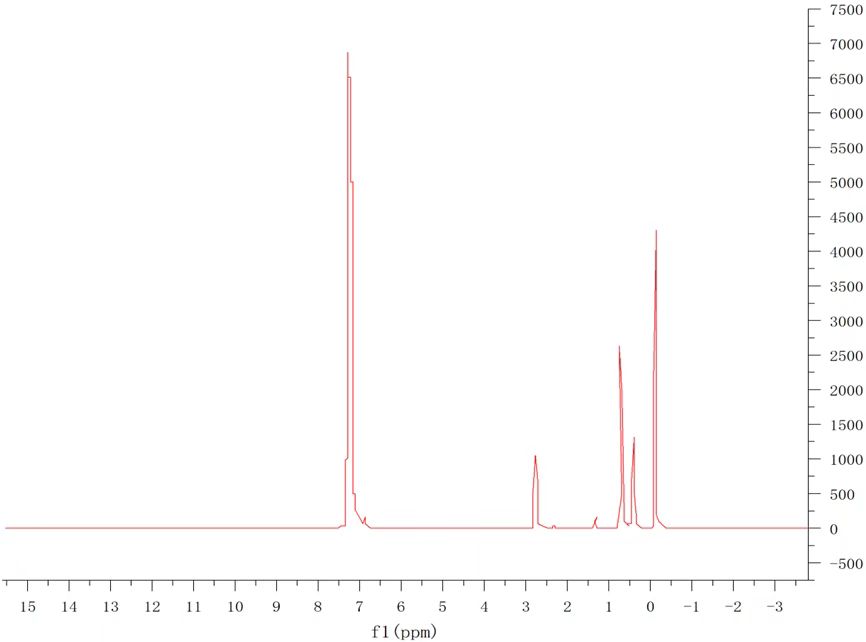
**

**(E)**

**Figure S1. ^1^H NMR spectra of quaternary phosphonium molecules**. (A) [MTPP]·I: ^1^H NMR (400 MHz, Chloroform-d) δ: 7.86-7.61 (m, 11H), 7.33 (s, 4H), 3.25 (dd, J = 13.2, 3.7 Hz, 3H); (B) [ETPP]·I：^1^H NMR (400 MHz, Chloroform-d) δ: 7.85-7.76 (m, 9H), 7.71 (d, J = 7.1 Hz, 6H), 3.74 (dd, J = 12.7, 6.7 Hz, 2H), 1.38 (dt, J = 19.8, 7.2 Hz, 3H); (C) [ITPP]·I：^1^H NMR (400 MHz, Chloroform-d) δ: 7.99-7.89 (m, 6H), 7.76 (d, J = 7.0 Hz, 3H), 7.70 (6, J = 7.6 Hz, 6H), 5.30-5.18 (m, 1H), 1.36 (d, J = 6.5 Hz, 3H), 1.31 (d, J = 6.4 Hz, 3H); (D) [BTPP]·I：^1^H NMR (400 MHz, Chloroform-d) δ: 7.82 (dt, J = 16.8, 8.2 Hz, 9H), 7.75-7.67 (m, 6H), 3.77-3.66 (m, 2H), 1.70 (q, 2H), 1.61 (d, J = 9.9 Hz, 2H), 0.92 (t, 3H); (E) [PTPP]·I: ^1^H NMR (400 MHz, Chloroform-d) δ:7.72–7.54 (m, 15H), 3.40 (td, J = 10.9, 9.0, 5.4 Hz, 2H), 1.55–1.30 (m, 4H), 1.16 (h, J = 7.2 Hz, 2H), 0.66 (t, J = 7.3 Hz, 3H).
